# Appropriate use criteria for echocardiography in the Netherlands

**DOI:** 10.1007/s12471-017-0960-9

**Published:** 2017-02-28

**Authors:** B. J. Bouma, R. Riezenbos, A. J. Voogel, M. H. Veldhorst, W. Jaarsma, J. Hrudova, B. Cernohorsky, S. Chamuleau, R. B. A. van den Brink, R. Breedveld, C. Reichert, O. Kamp, R. Braam, J. P. van Melle

**Affiliations:** 1 Department of Cardiology, AMC, Amsterdam, The Netherlands; 2 Department of Cardiology, OLVG, Amsterdam, The Netherlands; 3 Department of Cardiology, Spaarne Hospital, Hoofddorp, The Netherlands; 4 Department of Cardiology, Isala, Zwolle, The Netherlands; 5 Department of Cardiology, St. Antonius Hospital, Nieuwegein, The Netherlands; 6 Department of Cardiology, St Antonius Hospital, Sneek, The Netherlands; 7 Department of Cardiology, University Medical Center, Utrecht, The Netherlands; 8 Department of Cardiology, MCL, Leeuwarden, The Netherlands; 9 Department of Cardiology, MCA, Alkmaar, The Netherlands; 10 Department of Cardiology, VUmc and AMC, Amsterdam, The Netherlands; 11 Department of Cardiology, Gelre Hospitals, Apeldoorn, The Netherlands; 12 Department of Cardiology, University Medical Center Groningen, Groningen, The Netherlands

**Keywords:** Appropriate use criteria, Cardiac imaging, Diagnostic testing, Echocardiography

## Abstract

**Introduction:**

Appropriate use criteria (AUC) for echocardiography based on clinical scenarios were previously published by an American Task Force. We determined whether members of the Dutch Working Group on Echocardiography (WGE) would rate these scenarios in a similar way.

**Methods:**

All 32 members of the WGE were invited to judge clinical scenarios independently using a blanked version of the previously published American version of AUC for echocardiography. During a face-to-face meeting, consensus about the final rating was reached by open discussion for each indication. For reasons of simplicity, the scores were reduced from a 9-point scale to a 3-point scale (indicating an appropriate, uncertain or inappropriate echo indication, respectively).

**Results:**

Nine cardiologist members of the WGE reported their judgment on the echo cases (*n* = 153). Seventy-one indications were rated as appropriate, 35 were rated as uncertain, and 47 were rated as inappropriate. In 5% of the cases the rating was opposite to that in the original (appropriate compared with inappropriate and vice versa), whereas in 20% judgements differed by 1 level of appropriateness. After the consensus meeting, the appropriateness of 7 (5%) cases was judged differently compared with the original paper.

**Conclusions:**

Echocardiography was rated appropriate when it is applied for an initial diagnosis, a change in clinical status or a change in patient management. However, in about 5% of the listed clinical scenarios, members of the Dutch WGE rated the AUC for echocardiography differently as compared with their American counterparts. Further research is warranted to analyse this decreased external validity.

**Electronic supplementary material:**

The online version of this article (doi: 10.1007/s12471-017-0960-9) contains supplementary material, which is available to authorized users.

## Background

Sonography of the heart is one of the most widely used diagnostic tests in medicine. It has acquired a central role in the diagnosis, management, and follow-up of patients with any suspected or known heart diseases. The Dutch Working Group on Echocardiography (WGE) is an official working group within the Netherlands Society of Cardiology (NVVC) and one of its assignments is to guard the quality in clinical care, especially concerning echocardiography. The huge proportion of patients that nowadays undergo a first or follow-up echocardiographic evaluation in daily clinical care forced the WGE to search for tools to regulate the access to echocardiographic assessment according to clinical priority.

In 2011, the American College of Cardiology published a revision of appropriate use criteria (AUC) regarding transthoracic and transoesophageal echocardiography and stress echocardiography [1]. A total of 202 common clinical indications (clinical scenarios) for echocardiography were scored by an independent panel. The final results were formulated as the AUC for echocardiography. These criteria were thought to have a positive impact on physician decision-making, healthcare delivery, and reimbursement policy.

Several studies [2–7] have been published reporting the benefit of these AUC for echocardiography in reducing the amount of unnecessary cardiac echoes. However, external validity might be questioned as indications for echocardiography differ among hospitals within a country. Also, the organisation and implementation of healthcare differ strongly from country to country. Moreover, consensus documents are to a variable degree based on personal (i. e. expert) opinions instead of more quantitative (i. e. evidence-based) arguments for certain clinical management policies. Finally, although many scenarios were presented, several clinical conditions were not included, such as the interruption of anticoagulant therapy in patients with mechanical valves, patients with trauma, etc.

It is the aim of the current study to investigate whether the Dutch WGE would rate the clinical scenarios in a similar way as described in the original paper in order to determine the external validity.

## Methods

All 32 members of the WGE and members of the NVVC were invited to judge the 202 clinical scenarios of the original ACC paper [1] independently. All scenarios were judged using a version of the document in which the original ratings were blanked. They were asked to judge each clinical scenario based on their daily routine, expertise, and European Society of Cardiology practice guidelines relevant to the indications without cross checking with the original ACC manuscript. All responses were collected and analysed anonymously. During a separate face-to-face meeting in which panel members were provided with a blinded summary of their peers’ scores, consensus about the final rating was reached by open discussion for each indication. Due to the limited use of dobutamine stress echocardiography, leading to a limited number of experts who are able to judge the applicability in our country, the document was shortened by omitting 48 cases regarding limited used indications for stress echocardiography: Cases 119 to 154, and 163 to 175 were omitted from the final version. For reasons of simplicity, the scores were reduced from a 9-point scale to a 3-point scale referring to inappropriate (original ACC/AHA scale 1 to 3), uncertain (original ACC/AHA scale 4 to 6), and appropriate use criteria (original ACC/AHA scale 6 to 9).

## Results

Nine cardiologists and members of the Dutch WGE judged the 154 clinical scenarios (see Supplementary online data). Two out of nine worked in a university hospital, five in a hospital with cardiac surgery. Less than 1% of the answers were missing.

### Individual rating of the scenarios

**Fig. 1:**
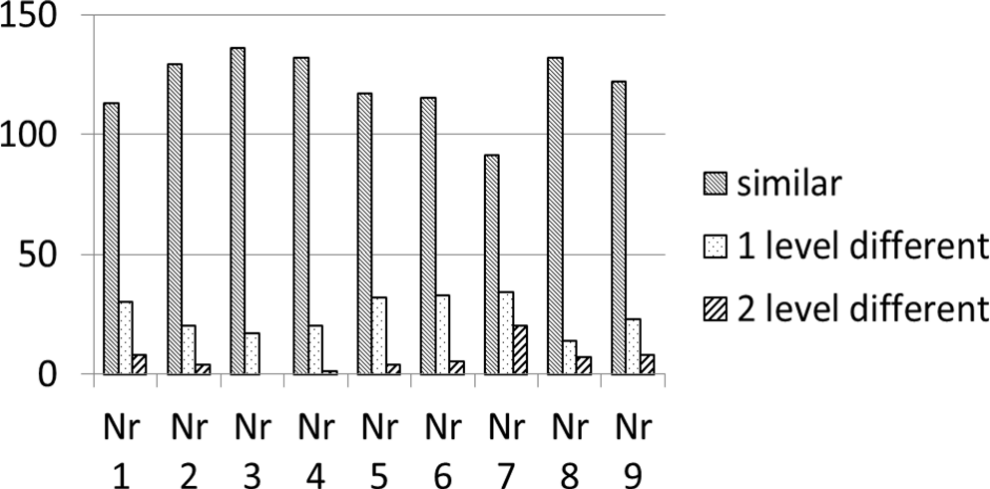
Frequency distribution of agreement and disagreement with the original AUC of the nine individual WGE members in comparison with the original rating. The Y‑axis shows the number of clinical scenarios assessed

In the original paper, 52 of the scenarios were rated as inappropriate use, compared with 47 by the individual Dutch cardiologists. Uncertain and appropriate use were rated in 35 and 71 scenarios by our panel (24 and 77 respectively in the original paper). The differences of the individual ratings compared with the original paper are presented in Fig. 1. In 64 cases the answer was the same for all cardiologists. In 65 cases there was at least 1 level of disparity in the answers, but the average answer of the nine cardiologists was similar to that in the original paper. In 24 cases the mean answer of the nine cardiologists together differed compared with the original paper (Table 1). Most differences were in the indications for stress echocardiography in scenarios evaluating ischaemia (2/5, 40%) and those for stress echocardiography for chronic valvular heart disease (6/25, 24%).

**Table 1:** Scenarios with a different judgement after the individual assessment

Scenario	Table	Rating in original paper	Mean rating on individual level	Mean rating after consensus meeting
8	1	1	2	1
9	1	3	2	3
20	2	2	3	3
28	2	1	2	1
30	2	1	2	1
35	3	1	2	2
38	3	1	2	1
40	3	1	2	1
56	3	1	2	2
66	5	1	2	1
68	6	1	2	1
77	6	2	3	2
98	7	2	3	2
99	8	3	2	3
104	8	3	2	3
110	8	2	1	1
115	9	3	2	3
116	9	3	2	3
184	17	2	1	2
185	17	3	2	2
187	17	2	1	1
188	17	3	2	2
194	17	2	1	2
195	17	3	2	3

1 = inappropriate, 2 = uncertain, 3 = appropriate

### Consensus meeting

The 24 cases in which the mean judgement differed from the original paper were discussed extensively during the consensus meeting. Finally, after the consensus meeting, in only 7 cases the appropriateness was judged differently than in the original paper (see Supplementary online data). These cases were the following. Case 20 was judged as appropriate instead of uncertain. The majority of the group thought that the assessment of volume status in a critically ill patient is a meaningful diagnostic test and often performed in their clinical practice. However, the US authors could be led in their rating by uncertainties as ‘how to best assess volume status’ as it is not always straightforward and is open to differences in interpretation. In case 35 the score was judged as uncertain instead of inappropriate as there was consensus that echo can contribute to patient care in the evaluation of an asymptomatic murmur or click, although routine echocardiography without physical examination is not justified. Case 56 was judged as uncertain instead of inappropriate as it was believed that restrictive use of echocardiography in such a serious disease would give the wrong signal. Case 110 was considered inappropriate because a non-cardiac source had already been determined. Subsequent transoesophageal echocardiography is not of additional value. In case 185, the indication for stress echocardiography in asymptomatic severe mitral regurgitation and a left ventricle not meeting the surgical criteria was considered uncertain (as opposed to appropriate in the ACC paper). In case 187, stress echocardiography for asymptomatic moderate aortic regurgitation, the score was judged as inappropriate (as opposed to uncertain in the ACC paper). In case 188 the indication for stress echocardiography for asymptomatic severe aortic regurgitation and a left ventricle not meeting surgical criteria was also rated as uncertain (as opposed to appropriate in the ACC paper). These last three cases all concern patients who are asymptomatic, and in whom the stress echo would not change clinical management. In case 104 a remark was added that it should not be used as the initial test as transoesophageal echocardiography is not indicated as the initial diagnostic test in suspected aortic pathology including but not limited to dissection/transsection, because transoesophageal echocardiography might be harmful due to an increase in blood pressure. In our opinion, CT or MRI should be the initial test to explore the possibility of aortic dissection.

In the Supplementary online data, the clinical scenarios are presented with the final judgement of the Dutch WGE.

## Discussion

This is the first paper in which cardiologists dedicated to echocardiography compare their opinion about appropriateness of echocardiography in clinical scenarios with that of well-respected panellists of the original AUC publication. In summary, and in line with our American colleagues, echocardiography was rated appropriate when it is applied for an initial diagnosis, a change in clinical status or a change in patient management. Routine testing or a test without change in management are more likely to be inappropriate. However, an important finding was that in about 20%, the individual rating scores of clinical scenarios were different as compared with the ratings in the original paper. In addition, differences in opinion were reflected by lengthy discussions in the consensus meeting and by the remaining differences compared with the original paper. This illustrates the variation in clinical practice among dedicated cardiologists.

The expanding use of imaging modalities in daily care and limited resources were the trigger to develop the AUC. The primary goal of the AUC presented in 2011 was to obtain a rational use of imaging services to deliver high-quality care [1]. These criteria will enable physicians to improve patient care and health outcomes in a cost-effective manner. However, differences in clinical practice are common between Europe and the USA as illustrated, for example, by the guidelines on valvular heart disease [8]. For example, stress echocardiography for asymptomatic moderate aortic regurgitation is not indicated according to the 2012 ESC guidelines on valvular heart disease [9]. We found at an individual level that in 5% of the cases, Dutch cardiologists gave the opposite rating on the appropriateness to that in the AUC paper. In 20% the rating differed by one level, i. e. inappropriate or appropriate instead of uncertain, or uncertain instead of inappropriate or appropriate. This variation might be explained by variation in clinical management, [10] insufficient external validity of the cases, lack of knowledge and implementation of guidelines, and local policies [11]. This emphasises the need for more evidence-based guidelines for the good adherence and improvement of care. Moreover, as echocardiography is not the sole imaging modality for assessment of cardiac function, other modalities and their specific advantages should be taken into account too [1, 12, 13].

The current appropriateness criteria cover the majority of echocardiograpic studies in a general and university hospital. The current findings show the applicability of these criteria for the clinical practice of most hospitals. The clinical scenarios are easy to read, well structured, and are straightforward to interpret. This makes them a tool for every day practice, and applicable for everyone in the field. Their use will improve the responsible use of healthcare resources, motivate technicians by making echoes meaningful and reduce the burden of investigations for patients. Of note, the category of ‘uncertain’ is used when the panellists thought there were insufficient clinical data available for a definitive categorisation or there was substantial disagreement regarding the appropriateness of that indication. Therefore the rating ‘uncertain‘ should not be used as grounds for denial of reimbursement. However, an ‘uncertain’ appropriateness criterion may point to an undeveloped area of scientific research.

Focused cardiac ultrasound is a simplified, clinician-performed application of echocardiography that is rapidly expanding in use, especially in emergency and critical care medicine. In 2014 clinical recommendations addressing focused cardiac ultrasound in clinical care were published [14, 15]. Although these recommendations were formulated well and similar to the current AUC echocardiography criteria, differences in interpretation, cultural aspects and local policies might play a role in their interpretation. Therefore, this should be the subject of future of investigation by the Dutch WGE.

This document is a complementary paper on the document on standard operating procedures of echocardiography from the WGE in the Netherlands in order to improve quality of care [16]. Moreover, it can be of help for physicians in their decisions to refer patients for echocardiography. This will finally lead to a better efficacy of echocardiographic laboratories, less unnecessary investigations for patients, a reduction in waiting time and a reduction in costs.

## Limitation

In the present study, the rating of the clinical scenarios in the final paper was reduced to a 3-point scale rating system instead of a 9-point rating system for reasons of simplicity. However, this might have led to less information and make the paper less sensitive than the original one. Nevertheless, in our opinion the current document is a good reflection of cardiology healthcare in the Netherlands and can improve clinical decision-making resulting in a more effective use of resources.

To improve the accuracy of our outcomes, 48 clinical scenarios on stress echocardiography in coronary artery disease were omitted because the use of this test for the purpose of ischaemia detection or viability is limited in our country. The current AUC for echocardiography are based on consensus statements making them subordinate to evidence-based recommendations. This type of recommendation evaluates all the information on a clinical subject in a systematic way by reviewing, rating, and synthesising the large amount of literature and then making an unbiased, evidence-based series of recommendations on clinical problems. In this manner, evidence-based recommendations yield a higher impact improvement of physician performance and patient outcomes.

## Conclusion

Echocardiography was rated appropriate when it is applied for an initial diagnosis, a change in clinical status or change in patient management. However, the appropriateness for echocardiography was rated differently from the original paper in 5% of the clinical scenarios. Further research is warranted to analyse causes of decreased external validity.

## Caption Electronic Supplementary Material

Clinical scenarios rated by the Dutch Working Group echocardiography
