# Supplementary material for: Appropriate use criteria for echocardiography in the Netherlands
Source: Neth Heart J. 2017 Feb 28;25(5):330–4. doi: 10.1007/s12471-017-0960-9 (PMC5405027; doi:10.1007/s12471-017-0960-9)
Supplement: Supplementary file 1 — Clinical scenarios rated by the Dutch Working Group echocardiography [file 12471_2017_960_MOESM1_ESM.docx]

**Supplementary file**

**TTE for general evaluation of cardiac structure and function**

| T1 |  |  |
| --- | --- | --- |
|  | **Suspected cardiac aetiology—general with TTE** |  |
| 1 | Symptoms or conditions potentially related to suspected cardiac aetiology including but not limited to chest pain, shortness of breath, palpitations, TIA, stroke, or peripheral embolic event | A |
| 2 | Prior testing that is concerning for heart disease or structural abnormality including but not limited to chest X-ray, baseline scout images for stress echocardiogram, ECG, or cardiac biomarkers | A |
| 3 | Infrequent APCs or infrequent VPCs without other evidence of heart disease | I |
| 4 | Frequent VPCs or exercise-induced VPCs | A |
| 5 | Sustained or nonsustained atrial fibrillation, SVT, or VT | A |
| 6 | Asymptomatic isolated sinus bradycardia | I |
|  | **Lightheadedness/presyncope/syncope with TTE** |  |
| 7 | Clinical symptoms or signs consistent with a cardiac diagnosis known to cause lightheadedness/  presyncope/syncope (including but not limited to aortic stenosis, hypertrophic cardiomyopathy, or HF) | I |
| 8 | Lightheadedness/presyncope when there are no other symptoms or signs of cardiovascular | U |
| 9 | Syncope when there are no other symptoms or signs of cardiovascular disease | A |
|  | **Evaluation of ventricular function with TTE** |  |
| 10 | Routine surveillance of ventricular function with known CAD and no change in clinical status or cardiac exam | I |
| 11 | Routine surveillance of ventricular function with known CAD and no change in clinical status or cardiac exam | I |
| 12 | Evaluation of LV function with prior ventricular function evaluation showing normal function (e.g., prior echocardiogram, left ventriculogram, CT, SPECT MPI, CMR) in patients in whom there has been no change in clinical status or cardiac exam | I |
|  | **Perioperative evaluation with TTE** |  |
| 13 | Routine perioperative evaluation of ventricular function with no symptoms or signs of cardiovascular disease | I |
| 14 | Routine perioperative evaluation of cardiac structure and function prior to noncardiac solid organ transplantation | U |
|  | **Pulmonary hypertension with TTE** |  |
| 15 | Evaluation of suspected pulmonary hypertension including evaluation of right ventricular function and estimated pulmonary artery pressure | A |
| 16 | Routine surveillance (<1 y) of known pulmonary hypertension without change in clinical status or cardiac exam | I |
| 17 | Routine surveillance (≥1 y) of known pulmonary hypertension without change in clinical status or cardiac exam | A |
| 18 | Re-evaluation of known pulmonary hypertension if change in clinical status or cardiac exam or to guide therapy | A |
|  |  |  |

T1 refers to table 1 in the original paper [1]

**TTE for cardiovascular evaluation in an acute setting**

| T2 |  |  |
| --- | --- | --- |
|  | **Suspected cardiac aetiology—general with TTE** |  |
| 19 | Hypotension or haemodynamic instability of uncertain or suspected cardiac aetiology | A |
| 20 | Assessment of volume status in a critically ill patient | A |
|  | **Myocardial ischaemia/infarction with TTE** |  |
| 21 | Acute chest pain with suspected MI and nondiagnostic ECG when a resting echocardiogram can be performed during pain | A |
| 22 | Evaluation of a patient without chest pain but with other features of an ischaemic equivalent or laboratory markers indicative of ongoing MI | A |
| 23 | Suspected complication of myocardial ischaemia/infarction, including but not limited to acute mitral regurgitation, ventricular septal defect, free-wall rupture/tamponade, shock, right ventricular involvement, HF, or thrombus | A |
|  | **Evaluation of ventricular function after ACS with TTE** |  |
| 24 | Initial evaluation of ventricular function following ACS | A |
| 25 | Re-evaluation of ventricular function following ACS during recovery phase when results will guide therapy | A |
|  | **Respiratory failure with TTE** |  |
| 26 | Respiratory failure or hypoxaemia of uncertain aetiology | A |
| 27 | Respiratory failure or hypoxaemia when a noncardiac aetiology of respiratory failure has been established | U |
|  | **Pulmonary embolism with TTE** |  |
| 28 | Suspected pulmonary embolism in order to establish diagnosis | I |
| 29 | Known acute pulmonary embolism to guide therapy (e.g., thrombectomy and thrombolytics) | A |
| 30 | Routine surveillance of prior pulmonary embolism with normal right ventricular function and pulmonary artery systolic pressure | I |
| 31 | Re-evaluation of known pulmonary embolism after thrombolysis or thrombectomy for assessment  of change in right ventricular function and/or pulmonary artery pressure | A |
|  | **Cardiac trauma with TTE** |  |
| 32 | Severe deceleration injury or chest trauma when valve injury, pericardial effusion, or cardiac injury are possible or suspected | A |
| 33 | Routine evaluation in the setting of mild chest trauma with no electrocardiographic changes or biomarker elevation | I |
|  |  |  |

T2 refers to table 2 in the original paper [1] and so on

**TTE for evaluation of valvular function**

| T3 | **Murmur or click with TTE** |  |
| --- | --- | --- |
| 34 | Initial evaluation when there is a reasonable suspicion of valvular or structural heart disease | A |
| 35 | Initial evaluation when there are no other symptoms or signs of valvular or structural heart disease | U |
| 36 | Re-evaluation in a patient without valvular disease on prior echocardiogram and no change in clinical status or cardiac exam | I |
| 37 | Re-evaluation of known valvular heart disease with a change in clinical status or cardiac exam or to guide therapy | A |
|  | **Native valvular stenosis with TTE** |  |
| 38 | Routine surveillance (<3 y) of mild valvular stenosis without a change in clinical status or cardiac exam | I |
| 39 | Routine surveillance (≥3 y) of mild valvular stenosis without a change in clinical status or cardiac exam | A |
| 40 | Routine surveillance (<1 y) of moderate or severe valvular stenosis without a change in clinical status or cardiac exam | U |
| 41 | Routine surveillance ((≥1 y) of moderate or severe valvular stenosis without a change in clinical status or cardiac exam | A |
|  | **Native valvular regurgitation with TTE** |  |
| 42 | Routine surveillance of trace valvular regurgitation | I |
| 43 | Routine surveillance (<3 y) of mild valvular regurgitation without a change in clinical status or cardiac exam | I |
| 44 | Routine surveillance (≥3 y) of mild valvular regurgitation without a change in clinical status or cardiac exam | U |
| 45 | Routine surveillance (<1 y) of moderate or severe valvular regurgitation without a change in clinical status or cardiac exam | U |
| 46 | Routine surveillance (≥1 y) of moderate or severe valvular regurgitation without change in clinical status or cardiac exam | A |
|  | **Prosthetic valve with TTE** |  |
| 47 | Initial postoperative evaluation of prosthetic valve for establishment of baseline | A |
| 48 | Routine surveillance (<3 y after valve implantation) of prosthetic valve if no known or suspected valve dysfunction | I |
| 49 | Routine surveillance (≥3 y after valve implantation) of prosthetic valve if no known or suspected valve dysfunction | A |
| 50 | Evaluation of prosthetic valve with suspected dysfunction or a change in clinical status or cardiac exam | A |
| 51 | Re-evaluation of known prosthetic valve dysfunction when it would change management or guide therapy | A |
|  | **Infective endocarditis (native or prosthetic valves) with TTE** |  |
| 52 | Initial evaluation of suspected infective endocarditis with positive blood cultures or a new murmur | A |
| 53 | Transient fever without evidence of bacteraemia or a new murmur | I |
| 54 | Transient bacteraemia with a pathogen not typically associated with infective endocarditis and/or a documented nonendovascular source of infection | I |
| 55 | Re-evaluation of infective endocarditis at high risk for progression or complication or with a change in clinical status or cardiac exam | A |
| 56 | Routine surveillance of uncomplicated infective endocarditis when no change in management is contemplated | U |
|  |  |  |

**TTE for evaluation of intracardiac and extracardiac structures and chambers**

| T4 |  |  |
| --- | --- | --- |
| 57 | Suspected cardiac mass | A |
| 58 | Suspected cardiovascular source of embolus | A |
| 59 | Suspected pericardial conditions | A |
| 60 | Routine surveillance of known small pericardial effusion with no change in clinical status | I |
| 61 | Re-evaluation of known pericardial effusion to guide management or therapy | A |
| 62 | Guidance of percutaneous noncoronary cardiac procedures including but not limited to pericardiocentesis, septal ablation, or right ventricular biopsy | A |
|  |  | A |

**TTE for evaluation of aortic disease**

| T5 |  |  |
| --- | --- | --- |
| 63 | Evaluation of the ascending aorta in the setting of a known or suspected connective tissue disease or genetic condition that predisposes to aortic aneurysm or dissection (e.g. Marfan syndrome) | A |
| 64 | Re-evaluation of known ascending aortic dilation or history of aortic dissection to establish a baseline rate of expansion or when the rate of expansion is excessive | A |
| 65 | Re-evaluation of known ascending aortic dilation or history of aortic dissection with a change in clinical status or cardiac exam or when findings may alter management or therapy | A |
| 66 | Routine re-evaluation for surveillance of known ascending aortic dilation or history of aortic  dissection without a change in clinical status or cardiac exam when findings would not change management or therapy | I |
|  |  |  |

**TTE for evaluation of hypertension, HF, or cardiomyopathy**

| T6 | **Hypertension with TTE** |  |
| --- | --- | --- |
| 67 | Initial evaluation of suspected hypertensive heart disease | A |
| 68 | Routine evaluation of systemic hypertension without symptoms or signs of hypertensive heart disease | I |
| 69 | Re-evaluation of known hypertensive heart disease without a change in clinical status or cardiac exam | U |
|  | **HF with TTE** |  |
| 70 | Initial evaluation of known or suspected HF (systolic or diastolic) based on symptoms, signs, or abnormal test results | A |
| 71 | Re-evaluation of known HF (systolic or diastolic) with a change in clinical status or cardiac exam without a clear precipitating change in medication or diet | A |
| 72 | Re-evaluation of known HF (systolic or diastolic) with a change in clinical status or cardiac exam with a clear precipitating change in medication or diet | U |
| 73 | Re-evaluation of known HF (systolic or diastolic) to guide therapy | A |
| 74 | Routine surveillance (<1 y) of HF (systolic or diastolic) when there is no change in clinical status or cardiac exam | I |
| 75 | Routine surveillance (≥1 y) of HF (systolic or diastolic) when there is no change in clinical status or cardiac exam | U |
|  | **Device evaluation (including pacemaker, ICD, or CRT) with TTE** |  |
| 76 | Initial evaluation or re-evaluation after revascularisation and/or optimal medical therapy to determine candidacy for device therapy and/or to determine optimal choice of device | A |
| 77 | Initial evaluation for CRT device optimisation after implantation | U |
| 78 | Known implanted pacing device with symptoms possibly due to device complication or suboptimal pacing device settings | A |
| 79 | Routine surveillance (<1 y) of implanted device without a change in clinical status or cardiac exam | I |
| 80 | Routine surveillance (≥1 y) of implanted device without a change in clinical status or cardiac exam | I |
|  | **Ventricular assist devices and cardiac transplantation with TTE** |  |
| 81 | To determine candidacy for ventricular assist device | A |
| 82 | Optimisation of ventricular assist device settings | A |
| 83 | Re-evaluation for signs/symptoms suggestive of ventricular assist device-related complications | A |
| 84 | Monitoring for rejection in a cardiac transplant recipient | A |
| 85 | Cardiac structure and function evaluation in a potential heart donor | A |
|  | **Cardiomyopathies with TTE** |  |
| 86 | Initial evaluation of known or suspected cardiomyopathy (e.g., restrictive, infiltrative, dilated,  hypertrophic, or genetic cardiomyopathy) | A |
| 87 | Re-evaluation of known cardiomyopathy with a change in clinical status or cardiac exam or to guide therapy | A |
| 88 | Routine surveillance (<1 y) of known cardiomyopathy without a change in clinical status or cardiac exam | I |
| 89 | Routine surveillance (≥1 y) of known cardiomyopathy without a change in clinical status or cardiac exam | U |
| 90 | Screening evaluation for structure and function in first-degree relatives of a patient with an inherited cardiomyopathy | A |
| 91 | Baseline and serial re-evaluations in a patient undergoing therapy with cardiotoxic agents | A |

**TTE for adult congenital heart disease**

| T7 |  |  |
| --- | --- | --- |
| 92 | Initial evaluation of known or suspected adult congenital heart disease | A |
| 93 | Known adult congenital heart disease with a change in clinical status or cardiac exam | A |
| 94 | Re-evaluation to guide therapy in known adult congenital heart disease | A |
| 95 | Routine surveillance (<2 y) of adult congenital heart disease following complete repair  + without a residual structural or haemodynamic abnormality  + without a change in clinical status or cardiac exam | I |
| 96 | Routine surveillance (≥2 y) of adult congenital heart disease following complete repair  + without residual structural or haemodynamic abnormality  + without a change in clinical status or cardiac exam | U |
| 97 | Routine surveillance (<1 y) of adult congenital heart disease following incomplete or palliative repair  + with residual structural or haemodynamic abnormality  + without a change in clinical status or cardiac exam | U |
| 98 | Routine surveillance (≥1 y) of adult congenital heart disease following incomplete or palliative repair  + with residual structural or haemodynamic abnormality  + without a change in clinical status or cardiac exam | U |
|  |  |  |

**TEE**

| T8 | **General uses** |  |
| --- | --- | --- |
| 99 | Use of TEE when there is a high likelihood of a nondiagnostic TTE due to patient characteristics or inadequate visualisation of relevant structures | A |
| 100 | Routine use of TEE when a diagnostic TTE is reasonably anticipated to resolve all diagnostic and management concerns | I |
| 101 | Re-evaluation of prior TEE finding for interval change (e.g., resolution of thrombus after anticoagulation, resolution of vegetation after antibiotic therapy) when a change in therapy is anticipated | A |
| 102 | Surveillance of prior TEE finding for interval change (e.g., resolution of thrombus after anticoagulation, resolution of vegetation after antibiotic therapy) when no change in therapy is anticipated | I |
| 103 | Guidance during percutaneous noncoronary cardiac interventions including but not limited to closure device placement, radiofrequency ablation, and percutaneous valve procedures | A |
| 104 | Suspected acute aortic pathology including but not limited to dissection/transsection (not as initial test) | A |
| 105 | Routine assessment of pulmonary veins in an asymptomatic patient status post pulmonary vein isolation | I |
|  | **Valvular disease** |  |
| 106 | Evaluation of valvular structure and function to assess suitability for, and assist in planning of, an intervention | A |
| 107 | To diagnose infective endocarditis with a low pretest probability (e.g., transient fever, known alternative source of infection, or negative blood cultures/atypical pathogen for endocarditis) | I |
| 108 | To diagnose infective endocarditis with a moderate or high pretest probability (e.g., staph bacteraemia, fungaemia, prosthetic heart valve, or intracardiac device) | A |
|  | **Embolic event** |  |
| 109 | Evaluation for cardiovascular source of embolus with no identified noncardiac source | A |
| 110 | Evaluation for cardiovascular source of embolus with a previously identified noncardiac source | I |
| 111 | Evaluation for cardiovascular source of embolus with a known cardiac source in which a TEE would not change management | I |
|  | **Atrial fibrillation/flutter** |  |
| 112 | Evaluation to facilitate clinical decision making with regard to anticoagulation, cardioversion, and/or radiofrequency ablation | A |
| 113 | Evaluation when a decision has been made to anticoagulate and not to perform cardioversion | I |
|  |  |  |

**Stress echocardiography for detection of CAD/Risk assessment: Symptomatic or ischaemic equivalent**

| T9 | **Evaluation of ischaemic equivalent (nonacute) with stress echocardiography** |  |
| --- | --- | --- |
| 114 | Low pretest probability of CAD  ECG interpretable and able to exercise | I |
| 115 | Low pretest probability of CAD  ECG uninterpretable or unable to exercise | A |
| 116 | Intermediate pretest probability of CAD  ECG interpretable and able to exercise | A |
| 117 | Intermediate pretest probability of CAD  ECG uninterpretable or unable to exercise | A |
| 118 | High pretest probability of CAD  Regardless of ECG interpretability and ability to exercise | A |
|  |  |  |

**Stress echocardiography for risk assessment: Perioperative evaluation for noncardiac surgery without active cardiac conditions**

| T13 | **Intermediate-risk surgery with stress echocardiography** |  |
| --- | --- | --- |
| 155 | Moderate to good functional capacity (≥4 METs) | I |
| 156 | No clinical risk factors | I |
| 157 | ≥ clinical risk factor  Poor or unknown functional capacity (<4 METs) | U |
| 158 | Asymptomatic <1 y post normal catheterisation, noninvasive test, or previous revascularisation | I |
|  | **Vascular surgery with stress echocardiography** |  |
| 159 | Moderate to good functional capacity (≥4 METs) | I |
| 160 | No clinical risk factors | I |
| 161 | ≥ 1 clinical risk factor  Poor or unknown functional capacity (<4 METs) | A |
| 162 | Asymptomatic <1 y post normal catheterisation, noninvasive test, or previous revascularisation | I |
|  |  |  |

**Stress echocardiography for assessment of viability/ischaemia**

| T16 | **Ischaemic cardiomyopathy/assessment of viability with stress echocardiography** |  |
| --- | --- | --- |
| 176 | Known moderate or severe LV dysfunction  Patient eligible for revascularisation  Use of dobutamine stress only | A |
|  |  |  |

**Stress echocardiography for haemodynamics (includes Doppler during stress) (former table 17)**

| T17 | **Chronic valvular disease—asymptomatic with stress echocardiography** |  |
| --- | --- | --- |
| 177 | Mild mitral stenosis | I |
| 178 | Moderate mitral stenosis | U |
| 179 | Severe mitral stenosis | A |
| 180 | Mild aortic stenosis | I |
| 181 | Moderate aortic stenosis | U |
| 182 | Severe aortic stenosis | U |
| 183 | Mild mitral regurgitation | I |
| 184 | Moderate mitral regurgitation | I |
| 185 | Severe mitral regurgitation  LV size and function not meeting surgical criteria | U |
| 186 | Mild aortic regurgitation | I |
| 187 | Moderate aortic regurgitation | I |
| 188 | Severe aortic regurgitation  LV size and function not meeting surgical criteria | U |
|  | **Chronic valvular disease—symptomatic with stress echocardiography** |  |
| 189 | Mild mitral stenosis | U |
| 190 | Moderate mitral stenosis | A |
| 191 | Severe mitral stenosis | I |
| 192 | Severe aortic stenosis | I |
| 193 | Evaluation of equivocal aortic stenosis  Evidence of low cardiac output or LV systolic dysfunction (‘low gradient aortic stenosis’)  Use of dobutamine only | A |
| 194 | Mild mitral regurgitation | U |
| 195 | Moderate mitral regurgitation | A |
| 196 | Severe mitral regurgitation  Severe LV enlargement or LV systolic dysfunction | I |
|  | **Acute valvular disease with stress echocardiography** |  |
| 197 | Acute moderate or severe mitral or aortic regurgitation | I |
|  | **Pulmonary hypertension with stress echocardiography** |  |
| 198 | Suspected pulmonary artery hypertension  Normal or borderline elevated estimated right ventricular systolic pressure on resting echocardiographic study | U |
| 199 | Routine evaluation of patients with known resting pulmonary hypertension | I |
| 200 | Re-evaluation of patient with exercise-induced pulmonary hypertension to evaluate response to therapy | U |
|  |  |  |

**Contrast use in TTE/TEE or stress echocardiography**

| T18 |  |  |
| --- | --- | --- |
| 201 | Routine use of contrast  All LV segments visualised on noncontrast images | I |
| 202 | Selective use of contrast  ≥2 contiguous LV segments are not seen on noncontrast images | A |
|  |  |  |

Reference

1. ACCF/ASE/AHA/ASNC/HFSA/HRS/SCAI/SCCM/SCCT/SCMR 2011 Appropriate Use Criteria for Echocardiography. J Am Soc Echocardiogr. 2011;24:229–67.
